# Orbital Metastases of Breast Cancer in Males

**DOI:** 10.3390/diagnostics13040780

**Published:** 2023-02-18

**Authors:** Cosmin Adrian Teodoru, Mihai Dan Roman, Horațiu Dura, Maria-Emilia Cerghedean-Florea

**Affiliations:** 1 Clinical Surgical Department, Faculty of Medicine, University “Lucian Blaga” Sibiu, 550169 Sibiu, Romania; 2 Department of Orthopedics and Trauma, Faculty of Medicine, University “Lucian Blaga” Sibiu, 550169 Sibiu, Romania; 3 Preclinical Department, Faculty of Medicine, University “Lucian Blaga“ Sibiu, 550169 Sibiu, Romania

**Keywords:** male breast cancer, orbital metastases, infiltrating breast carcinoma

## Abstract

We report a case of orbital metastasis of infiltrative breast carcinoma in a 65-year-old man. The patient was diagnosed with stage four breast cancer one year before, for which a mastectomy was performed. He refused postoperative radiotherapy and chemotherapy at that time. He had a history of lung, liver, and mediastinal metastases. At admission, he presented with blurred vision, diplopia, ocular pain, and mild swelling of the upper lid of the left eye (LE). Computed tomography (CT) of the brain and orbit revealed a front-ethmoidal tissue mass with left orbital and frontal intracranial extension. Ophthalmologic examination revealed exophthalmos on the LE with a downward and outward deviation of the eyeball, proptosis, and intraocular pressure (IOP) of 40 mmHg. The patient’s treatment started with topical maximal anti-glaucomatous drops and radiotherapy sessions. After three weeks of follow-up, there was a gradual improvement of local symptoms and signs and a normal IOP.

**Figure 1:**
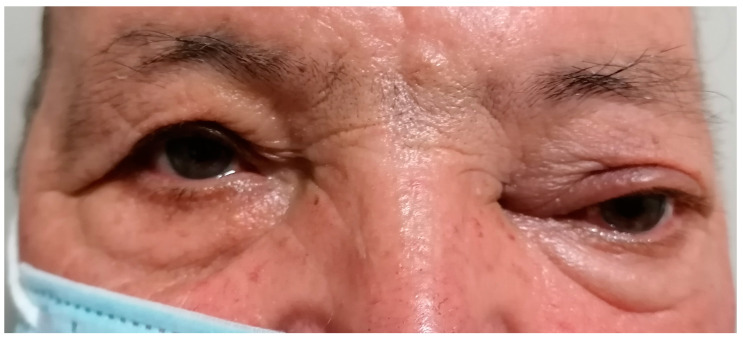
A 65-year-old male presented with blurred vision, diplopia, ocular pain, and a slight swelling of the upper eyelid on the left eye. His medical history was remarkable for an infiltrating breast carcinoma (one year ago) for which he had a mastectomy. Ophthalmic examination showed exophthalmos on LE with eyeball deviation downwards and outwards with proptosis. The best-corrected acuity vision (BCVA) was 1 for the right eye (RE) and 0.9 for LE. Intra-ocular pressure (IOP) was 19 mmHg for RE and 40 mmHg for LE. The anterior segment for both eyes (OU) was normal. Extra-ocular movement of the left eye was limited in all directions. Examination of the posterior segment under dilatation on LE showed optic atrophy with the pallor of the optic nerve head, and a foveal reflex faded. The posterior segment of RE was normal. Breast cancer is one of the most common oncological pathologies diagnosed in women, being the second cause of death after lung cancer [1]. In contrast, breast cancer is very rare in men, accounting for about 1% of all diagnosed neoplasms [2,3]. Despite this, it is often diagnosed in late stages [4]. Ocular metastases of breast cancer, although quite rarely diagnosed, can occur in both women and men with an incidence according to different studies between 5–30% [5,6,7]. The histopathological examination revealed an invasive mammary carcinoma, grade 2 of malignancy, with lymphatic, vascular, and perineural invasion. He refused postoperative radiotherapy and chemotherapy. Additionally, he had a history of lung, liver, and mediastinal metastases. Immunohistochemical tests showed a positive result for estrogen receptor (ER)-80%, E-cadherin expression (ECAD), human epidermal growth factor receptor 2 (HER2) Ki67, and negative for progesterone receptors (PR). A standardized B-scan of the left eye did not reveal the presence of any intraocular mass (Figure 2).

**Figure 2:**
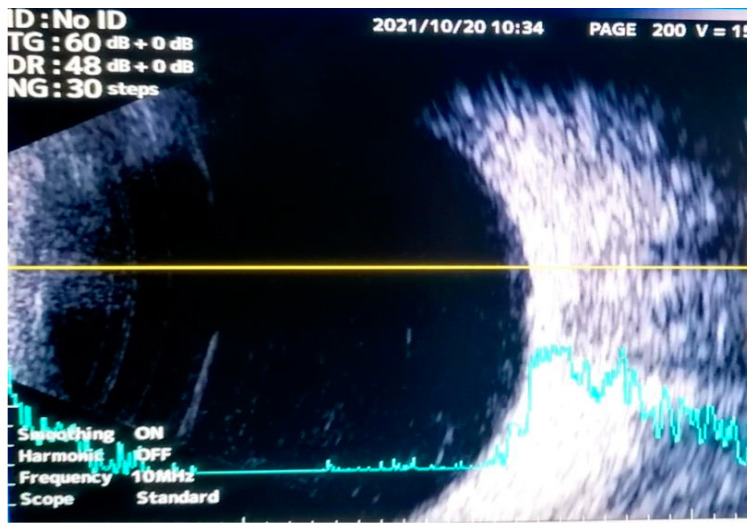
Standardized B-scan ultrasonography of the left eye did not reveal the presence of any intraocular mass. Computed tomography (CT) of the brain and orbit showed a front-ethmoid tissue mass with bone destruction and regional invasion (Figure 3).

**Figure 3:**
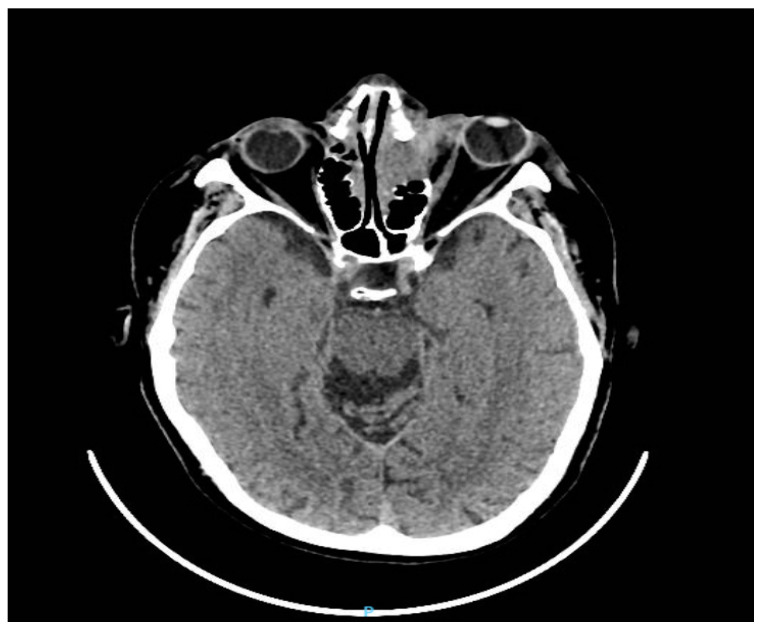
Computed tomography of the brain and orbit (axial planes) showed a front-ethmoid tissue mass with bone destruction and regional invasion with the left orbital extension in contact with the eyeball and left frontal intracranial extension of approximately 3.8/4/4.2 cm. The patient’s treatment began with maximal topical anti-glaucoma eye drops (topical beta-blockers, alpha-agonists, and carbonic anhydrase inhibitors). The patient’s care was subsequently co-managed with the oncological team. He started radiotherapy sessions according to the Intensity Modulated Radiation Therapy (IMRT)) scheme with a total dose of 30 Gy in 15 fractions given in 3 weeks and 2Gy/fraction. The irradiation was done with a thermoplastic mask after a CT planning where the tumor mass and adjacent structures were contoured (red color). Radiation doses delivered to normal anatomical structures (optic nerves, the lens) were within parameters (Figure 4).

**Figure 4:**
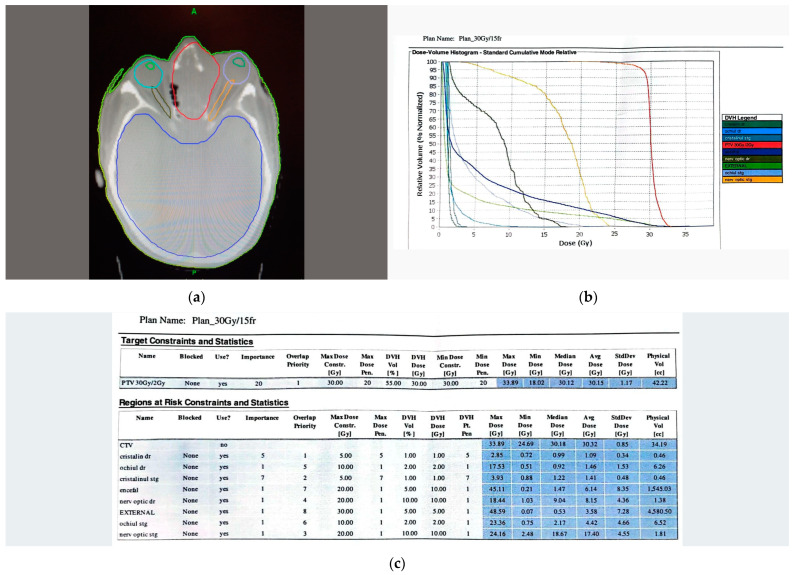
(**a**) Thermoplastic mask after a CT planning. The tumor mass and adjacent structures were contoured (red color). (**b**) Dose Volume Histogram. (**c**) Snapshot of the treatment plan. At three weeks of follow-up, after the radiotherapy sessions, the patient’s condition slightly improved and the IOP was normal under treatment (Figure 5). The patient remained under oncological supervision.

**Figure 5:**
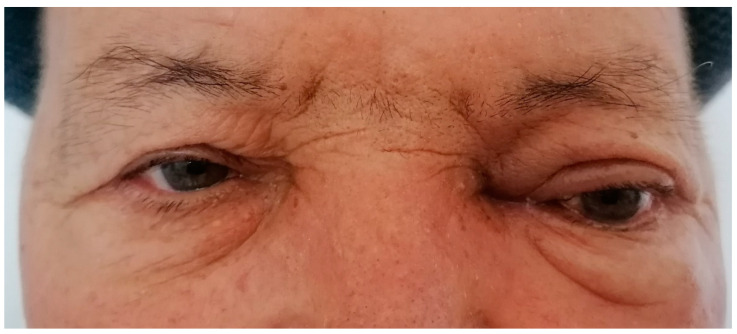
Left eye at one-month follow-up after treatment with maximal topical anti-glaucoma eye drops (topical beta-blockers, alpha-agonists, and carbonic anhydrase inhibitors) followed by radiotherapy sessions. We noticed a slight improvement in the symptomatology, with the disappearance of diplopia and normalization of intraocular pressure. Breast cancer in men (MBC) is very rare. Studies show that the incidence rate has increased with age. The risk of breast cancer in men is 1:1000 compared to 1:8 in women, but it doubles in men who have a first-degree relative diagnosed with breast cancer [8,9,10]. The prognosis for breast carcinoma in men is generally considered to be poorer than that for breast cancer in women and is usually discovered in the advanced stages of the disease [11,12]. The most important risk factors in the development of MBC are represented by hormonal imbalances and environmental conditions [13,14]. Due to the very low incidence, male breast cancer has been routinely excluded from breast cancer clinical trials. Usually, diagnostic and treatment recommendations for male breast cancer have been based on the results of clinical research focusing primarily on women. Mutations in BRCA are among the most clearly established risk factors for breast cancer in men. Recent studies from the literature have revealed clinically important differences in the carcinogenesis of male breast cancer, particularly related to the main susceptibility genes BRCA1 and BRCA2 highlighting the importance of comprehensive germline genetic testing in targeted, risk-adjusted surveillance programs for early cancer detection and targeted cancer therapy. It proves that there is a high prevalence of pathogenic variants of these genes, especially in the BRCA2 This leads to an increased risk of developing breast cancer compared to the general male population [15,16,17]. Unfortunately, genetic testing could not be performed in our case, due to the patient refusing it. Orbital metastases represent a low percentage of all orbital tumors with an incidence between 1–13% [18]. In terms of starting point, breast cancer is the most involved, closely followed by lung, gastrointestinal, kidney, prostate, or skin cancer [19,20,21,22,23]. Orbital metastases usually occur in patients with an established diagnosis of disseminated cancer and there is a long median time interval of 4.5–6.5 years from diagnosis for breast carcinoma [18]. In our case, orbital metastases occurred approximately 1 year after diagnosis and mastectomy. One main reason could be breast cancer prognosis and survival rates are lower in male patients [24]. Another issue is patient compliance and refusal of postoperative chemotherapy and radiotherapy. One of the most common symptoms of orbital metastases is diplopia. This is explained by the fact that there is an affinity of breast cancer-specific tissue for the extraocular muscle and surrounding orbital fat. Other common symptoms and signs found in our case are proptosis, eyelid swelling, pain, eyelid ptosis, and blurred vision due to infiltration or compression [25]. Increased intraocular pressure may be due, in the cases of intraorbital metastases, to a disturbance of the pressure gradient between intraocular pressure and pressure in the episcleral veins. The main mechanism, in this case, is direct compression or tumor invasion, causing secondary glaucoma [26]. Treatment for orbital metastases is unfortunately palliative, as the hematogenous spread of cancer to the orbit is a sign of systemic disease and involvement of other sites. Surgical intervention may be useful for palliative purposes (tumor resection or enucleation) in cases of unmanageable local symptoms [19]. In the approach of these cases, radiotherapy is an affordable option with high rates (60–80%) of clinical improvement in local symptoms and vision [23]. In our case, the only treatment was topical antiglaucoma eye drops combined with radiotherapy sessions. The response was satisfactory with a slow reduction of ocular symptoms and normalization of intraocular pressure under treatment. Despite this, the treatment is strictly for the remission of symptomatology, the results are visible in the short term and inconsistent, without solving the underlying problem. The occurrence of breast cancer in male patients is rare, so the diagnosis of orbital metastases from this cause is even more rare. However, ocular involvement should be suspected in all cases of ocular symptoms, especially in those with a history of oncological pathology. The therapeutic approach, as well as the response to treatment, depends on the stage of the disease and the patient’s compliance. In this case, the lack of postoperative chemotherapy and radiotherapy led to disease progression, with orbital metastatic dissemination one year later.

## Data Availability

Not applicable.
